# Wild vertebrates and their representation by urban/rural students in a region of northeast Brazil

**DOI:** 10.1186/s13002-018-0283-y

**Published:** 2019-01-05

**Authors:** José Valberto de Oliveira, Sérgio de Faria Lopes, Raynner Rilke Duarte Barboza, Dilma Maria de Melo Brito Trovão, Maiara Bezerra Ramos, Rômulo Romeu Nóbrega Alves

**Affiliations:** 1 Departamento de Biologia e Programa de Pós-Graduação em Etnobiologia e Consevação da Natureza, Universidade Estadual da Paraíba, Av. das Baraúnas, 351/Campus Universitário, Bodocongó, Campina Grande-PB, 58109-753 Brazil; 2 Escola Agrotecnica EAGRO, Universidade Federal de Roraima, Rodovia BR 174, Km 37, s/n – P.A. Nova Amazônia, Campus Murupu, Boa Vista-RR, 69.300-000 Brazil

**Keywords:** Animal recognition, Education, Biological literacy, Ethnozoology

## Abstract

**Background:**

Recognition of the diversity of living beings, including the classification and naming of species, is a fundamental condition for biological literacy with the aim of developing critical awareness of human relationships with nature, and for which formal education plays an important role. The present study aimed to analyze the representation that urban/rural students have for wild vertebrates and their main sources of knowledge.

**Methods:**

Data collection took place in three public schools, one urban and two rural, in the municipality of Campina Grande, Paraíba, Brazil. Questionnaires were given to 990 students (528 urban and 462 rural), distributed among all the grades that comprise middle school (Ensino Fundamental II, grades 6–9) and high school (Ensino Médio, grades 10–12) education.

**Results:**

A total of 5877 citations were mentioned by the students, which corresponded to 224 distinct animals with 166 (72.0%) being wild vertebrates, 24 (25.7%) being domestic vertebrates, and 34 (2.3%) being invertebrates. Mammals and reptiles had the greatest observed richness of citations, while mammals (*H*′ = 3.37), birds (*H*′ = 2.84), and invertebrates (*H′* = 2.94) had the greatest diversity. Positive correlations were found between citations of wild vertebrates and family income (rt = 0.06; *P* < 0.05) and curricular development (rs = 0.08; *P* < 0.01); negative correlations were found between curricular development and citations of domestic animals (rs = − 0.22; rs = − 0.11 *P* < 0.01) and between age group and citations of invertebrates (*r* = − 0.14; rs = − 0.11 *P <* 0.01). As for the sources of knowledge regarding the animals indicated by the students, “media,” “daily experiences,” “tradition” (here understood as knowledge resulting from interactions with parents and experienced community members), and “formal education” stood out.

**Conclusions:**

Comprehension of vertebrate diversity is a fundamental condition for the development of attitudes compatible with its conservation, which emphasizes the importance of biological literacy in achieving this purpose.

## Background

Knowledge of the diversity of living beings and, above all, the recognition of the importance of each species for ecological sustainability is a fundamental condition for the development of human behaviors and attitudes compatible with the conservation of nature [1]. Knowledge about biodiversity, made explicit by means of the naming of species, constitutes an indication of connectivity between humans and their immediate environment [2, 3] and contributes to the development of subsequent learning [4]. Knowing or identifying a unit of diversity presupposes, minimally, how to classify, from the initial grades of schooling, animals into groups such as vertebrates and invertebrates, and wild and domestic, as well as their ecological implications [5, 6]. Previous studies have recognized that in some cases children do not recognize these categories [7] and that children and adults have better recognition of domestic animals [3, 8]. Therefore, investments in early childhood education are fundamental to the appreciation of wildlife in subsequent stages of life [2, 4, 8]. In this sense, emphasis is on the role of formal biological education for enabling the comprehension of these initial categorizations, as processes of biological literacy, for fundamental changes in behaviors regarding issues of biodiversity [3, 4, 9–11].

Wild vertebrates constitute a group of organisms perhaps most directly involved with human evolutionary history, having been elements of diverse antagonistic interactions ranging from their use as a nutritional source and domestication for different purposes, to situations of conflict due to losses, predation, or accidents [12–14]. These interactions had repercussions in determining cultural patterns of affinity or aversion, depending on the vertebrate and its relation with people in each context [2, 15–17], thereby guiding behaviors and consequent attitudes towards animal conservation [18]. In this context, studies have recognized a tendency for greater affinity for large vertebrates that are showy in appearance, utilitarian, and charismatic [1, 3, 7, 8], including representatives of mammals, birds, and fish [2, 15], and, inversely, aversion to less showy, “unpleasant-looking” animals, seen as being useless or harmful to humans, including mainly representatives of amphibians and reptiles [15, 19–21]. This tendency generally leads to conservation projects that emphasize “lovable” species, especially mammals and birds, thus neglecting other less charismatic animals [10, 22], such as representatives of reptiles and amphibians [20].

Recognized means of acquiring knowledge about animals, especially vertebrates, include parental interactions [19, 23, 24]; direct interactions with species in everyday situations [13, 17], including activities such as fishing, feeding animals, and observing wildlife [25]; fictional stories [26]; access to the media [22, 26, 27]; non-formal education strategies such as museums, parks, and zoos; and, especially, access to formal education [3, 20, 25, 28] including images of animals in textbooks [29].

It is also important to consider that knowledge about the diversity of life presupposes interests and motivations, aspects related to, among other factors, socioeconomic variables such as income, gender, religion, age group, schooling, and place of residence [1, 3, 13, 16–18, 30–32]. Several studies have shown that the location of the dwelling place (rural or urban) influences interests and motivations directed towards fauna and its conservation [2, 15, 25, 26], including, among other determining factors, the education processes experienced. That is, broader formal, informal, and cultural educational processes make the relationships between humans and animals unequal between urban and rural realities, which are conditioned by several factors including the unique socio-cultural specificities of each context [3, 7, 18, 30]. From this perspective, Pinheiro et al. [13] emphasizes that the processes of schooling and media access for rural students are generally less efficient when compared to urban students. On the other hand, rural students generally have greater interaction with nature than urban school students [32].

However, the challenge is to educate the importance of the unity of diversity, with an emphasis on conservation, and guide behaviors and attitudes in human relationships with wild vertebrates [30]. In the course of this process, to reiterate, rather than simply recognizing and classifying animals, it is important to develop a critical awareness of the role of each in nature. In this context, formal biological education plays a fundamental role because of its function and objectives advocated by curricular guidelines [5, 6, 33]. Linked to this perspective is the fact that “biological illiteracy” is a worrisome factor in relation to conservation, since it limits the possibilities of developing initiatives with citizen participation for the conservation of local species [10], which should involve not only students, but also teachers and other educators—“key individuals” in the process [17, 23]. In short, limitations in basic knowledge of ecological and systematic aspects of species contribute significantly to human alienation from nature and its conservation [2], since knowledge is a fundamental condition for the development of positive attitudes [34], without which conservation efforts become useless [20].

In view of the above, the present study, developed in an area of the semi-arid region of Brazil, aimed to analyze the representation by urban/rural students about wild vertebrates and what are the main sources from which knowledge about these animals is derived. In this sense, the research was guided by the following questions: (1) What do students cite as wild vertebrates? (1.1) Do these representations differ between students from urban and rural areas? (2) In terms of richness and diversity, what is the representation landscape of the large groups of vertebrates cited? (3) Do variables such as gender, age, family income, religious orientation, and student curricular development influence representations of wild vertebrates? (4) What are the origins of knowledge about the wild vertebrates cited by the students?

## Methods

### Study area

**Fig. 1:**
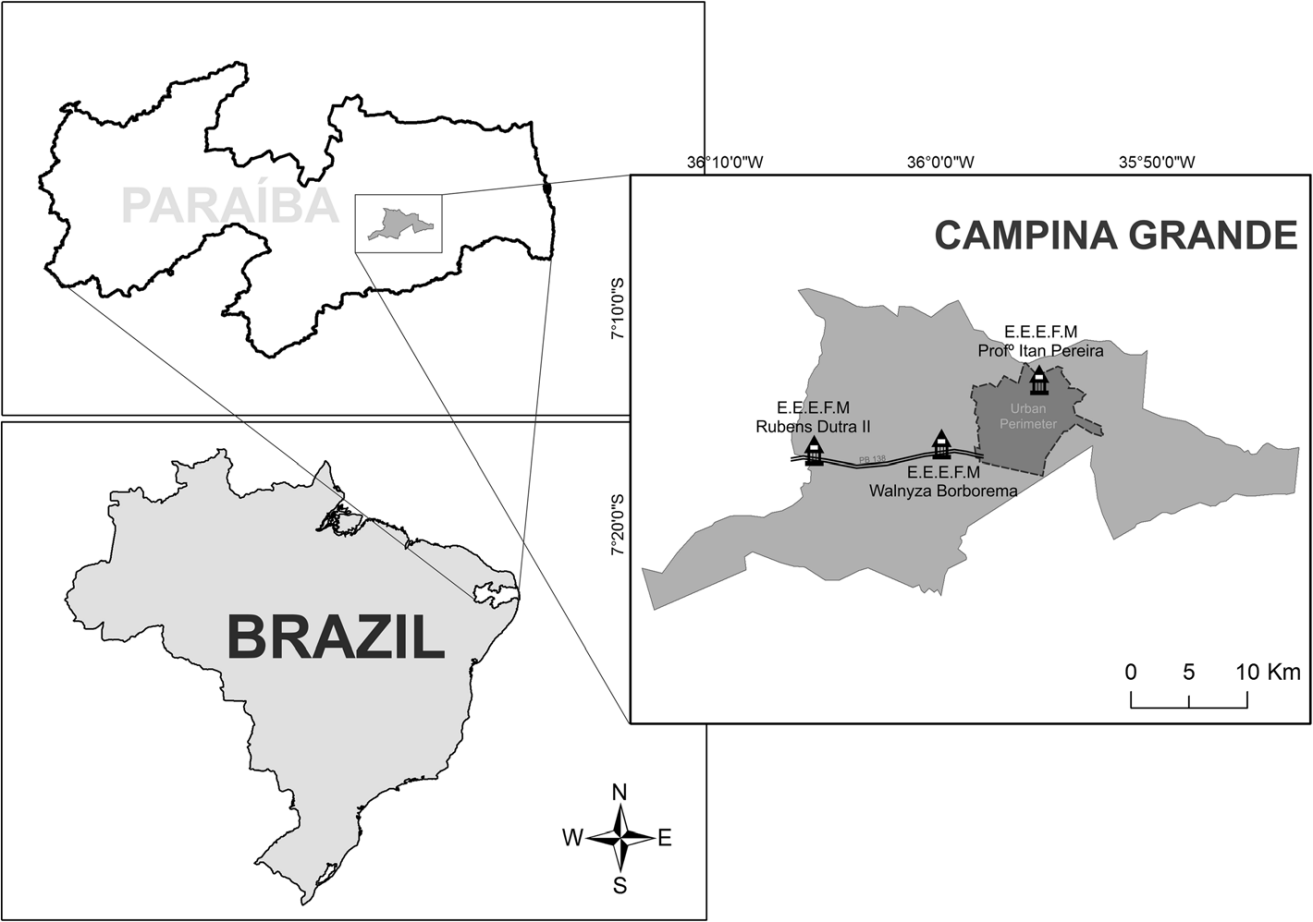
Map showing the locations of the studied schools of the municipality of Campina Grande, Paraíba

The research involved three schools, one urban and two rural, of the Rede Estadual (State Education Network) in the municipality of Campina Grande (07° 13′ 50″ S, 35° 52′ 52″ W), Paraíba, Northeast Brazil (Fig. 1). The municipality of Campina Grande has an area of 593,026 km^2^ and a population of 385,213 inhabitants, with 367,209 urban and 18,004 rural, giving it a population density of 648.31 inhabitants/km^2^. The Human Development Index for the municipality (HDI) is 0.720 [35].

The studied schools were selected according to the criterion of the presence of complete middle school (Ensino Fundamental II, grades 6–9) and high school (Ensino Médio, grades 10–12) levels of basic education. It should be emphasized that, in this municipality, the education of students from rural areas is concentrated in schools located at strategic points (e.g., road margins), where student access is optimized through public school transportation. It should also be emphasized that the inclusion of two schools aimed at rural students was done in order to equate the urban/rural sample “*n*”.

In view of the criteria described above, the following schools were included in the study: (1) Escola Estadual de Ensino Fundamental e Médio Professor Itam Pereira, located in an urban area in the west zone of the municipality and created by Decree no. 21.039/2000; (2) Escola Estadual de Ensino Fundamental e Médio Walnyza Borborema Cunha Lima, located in Sítio Estreito, 12 km west of the center of the municipal seat, with access by highway BR 230, and created by Resolution 36.730/2006/2016; and (3) Escola Estadual de Ensino Fundamental e Médio Rubens Dutra Segundo, located in Distrito de Catolé de Boa Vista, 26 km west of the municipal seat, with access by highway BR 230, and created by Decree 13.151/1989 (Fig. 1). At the time of the survey these schools had, respectively, 942, 444, and 328 students enrolled in regular school and special programs. Of these, we included in the study all those enrolled in the seven grades corresponding to the last two regular cycles of basic education: 6th to 9th grade middle school (Ensino Fundamental II) and 10th to 12th grade high school (years 1 to 3 Ensino Médio).

### Methodological procedures

#### Data collection

Data were collected by means of semi-structured questionnaires given to 990 students, of which 528 were urban and 462 rural. The ages of the respondents ranged from 9 to 38 years, with 464 being male and 526 being female. The questionnaires were given from June to October 2015 in Science/Biology classes and involved 24 middle school (Ensino Fundamental II) classes and 14 high school (Ensino Médio) classes. The questions asked the students to express their understanding of wild vertebrates by citing examples of animals, as well as their respective sources of knowing and/or knowledge of these animals. For the purpose of analysis, cited animals were categorized according to order (Linnaean Classification) or in broader taxa, when classification to order was not possible (Table 1).

**Table 1:** Categories of the origin of knowledge of animals and the defining criteria

Categories	Defining criteria
Tradition (Tr)	Content that refers to knowledge about animals through parents and experienced community members
Daily experiences (DE)	Knowledge from own experiences in immediate situations of exploration in the context of life
Field experiences (FE)	Knowledge through planned situations in natural environments: trails, camps and excursions
Pet (P)	Knowledge derived from experiences with animals raised in a domestic environment or captivity
Hunting/fishing (HF)	Knowledge through the practice of hunting and fishing
Trade (T)	Knowledge acquired in commercial situations such as open fairs and markets
Formal education (FoE)	Knowledge related to schooling: Science and Biology classes, didactic and para-didactic books, and extracurricular activities
Media (M)	Knowledge obtained through various technological resources: internet, TV programs, documentaries, and films
Zoological units (Z)	Knowledge acquired through zoos, oceanariums, aquariums, and zoonosis centers
Other (O)	Not in the categories above

Data collection was proceeded by the following ethical/legal procedures: research approval by the Comitê de Ética em Pesquisa of the Universidade Estadual da Paraíba (Protocol CEP-UEPB: 43589815.0.0000.5187), authorization by the administrative bodies responsible for the respective schools, agreement of Science/Biology teachers to collaborate with the study, and the presentation of research purposes to students and the sending of “Termos de Consentimento Livre e Esclarecidos – TCLs”, an ethical/legal requirement to effectively participate in the research process, to their parents and/or legal guardians. Only with the return of the duly signed TCLs did the data collection begin.

#### Data analysis

Descriptive data referring to the origins of knowledge about the cited animals were categorized according to the method of “content analysis” [36, 37], using the technique called for “acervo” (collection). In this categorization process, the contents of the messages emitted by the research participants were grouped by semantic criteria [37]. As a result, 10 categories referring to the origins of knowledge about the cited animals were obtained (Table 1).

In order to calculate the richness and diversity of the species cited by the students, 990 interviews were used randomly in the course of curricular evolution, i.e., the interviews were related to the students’ knowledge about wild animals, independent of the taxonomic group. Subsequently, the data were organized according to the citations of the species mentioned by the students in the five large vertebrate groups: fish, amphibians, reptiles, birds, and mammals.

Values for richness of each group were calculated for the cited animals. In addition, values for diversity^1^ of the cited animals for each group were calculated by adapting the equation of the Shannon-Weaver Index (*H′*) [38], where *ni* = number of citations for the *i*th animal; *N* = total number of citations; *S* = total number of animals cited; and ln = natural (Naperian) logarithm.

$$ {H}^{\hbox{'}}=\frac{\left[N1n(N)-\sum \limits_{i=1}^S{n}_i\ln \left({n}_i\right)\right]}{N} $$

To estimate the total number of species cited by group, we used the nonparametric Chao first-order estimator, which is capable of estimating total species richness from observed richness data, with 1000 randomizations. This analysis was performed using EstimateS© version 8.2 software [39].

Subsequently, non-parametric descriptive statistics were used to analyze the obtained data. For this, data were initially tested for normality by the Shapiro-Wilk test and for homoscedasticity using the Levene test. The general data were organized into percentages (tables) and box plot graphs were generated using the program Past 2.17c [40].

In order to evaluate differences between the number of citations for wild, domestic, and invertebrate animals, as well as to assess whether school level (middle school or high school) and religion influence the frequencies of citations of vertebrates, the Kruskal-Wallis H-test was used [41]. In order to assess the influence of income of participants in relation to the citations of wild vertebrates, we used Kendall’s tau coefficient. To evaluate the influence of age and school level of the participants in relation to citation frequencies of wild, domestic, and invertebrate animals, as well as the relation of the origins of knowledge with curricular development, Spearman correlations were performed for non-parametric tests. The statistical tests were performed with the program Past 2.17c [40].

## Results

### Understanding of wild vertebrates among urban and rural students: cited animals and influences of socioeconomic variables

Expressing their understanding of wild vertebrates, the students (*n* = 990) indicated 5877 citations referring to 224 animals. Of these, 72.0% corresponded to 166 wild animals, 25.7% corresponded to 24 domestic animals, and 2.3% to 34 invertebrates. Statistically significant differences were observed for citations of wild, domestic, and invertebrate animals (*H′* = 1007; *P* < 0.001) (Table 2). Of all the citations, 52.40% were from urban students and 47.59% from rural students. Among the citations by urban students, 80.0% corresponded to 132 wild animals, 18.7% to 21 domestic animals, and 1.3% to 17 invertebrates. Among the citations by rural students, 63.1% corresponded to 130 wild animals, 33.4% to 21 domestic animals, and 3.5% to 30 invertebrates.

**Table 2:** Frequencies (%) of animals cited per groups, by the research participants

Groups	Taxa	Animal^a^	General	Urban	Rural
Fishes	Fish	Peixe	144 (66.97)	77 (59.68)	67 (77.90)
	Tetraodontiformes	Baiacu	1 (0.46)	1 (0.77)	0 (0.00)
	Perciformes	Atum	1 (0.46)	1 (0.77)	0 (0.00)
		Tilápia	3 (1.39)	1 (0.77)	2 (2.32)
	Selachimorpha*	Tubarão	17 (7.90)	9 (6.97)	8 (9.30)
	Gasterosteiformes	Cavalo-marinho	1 (0.46)	0 (0.00)	1 (1.16)
	Osteoglossiformes	Pirarucu	2 (0.93)	2 (1.55)	0 (0.00)
		Piranha	18 (8.37)	15 (11.62)	3 (3.48)
		Traíra	9 (4.18)	7 (5.42)	2 (2.32)
		Curimatã	6 (2.79)	6 (4.65)	0 (0.00)
	Characiformes	Piaba	1 (0.46)	1 (0.77)	0 (0.00)
	Clupeiformes	Sardinha	9 (4.18)	7 (5.42)	2 (2.32)
	Siluriformes	Chupa pedra	1 (0.46)	1 (0.77)	0 (0.00)
	Salmoniformes	Salmão	1 (0.46)	1 (0.77)	0 (0.00)
	Cypriniformes	Carpa	1 (0.46)	0 (0.00)	1 (1.16)
Total	10	15	215 (99.93)	129 (99.63)	86 (99.96)
Amphibians	Amphibian	Anfíbio	1 (0.28)	1 (0.43)	0 (0.00)
	Anura	Sapo	170 (48.43)	103 (44.58)	67 (55.83)
		Cururu	2 (0.56)	0 (0.00)	2 (1.66)
		Sapo boi	1 (0.28)	0 (0.00)	1 (0.83)
		Rã	89 (25.35)	61 (26.40)	28 (23.33)
		Perereca	53 (15.09)	38 (16.45)	15 (12.50)
		Gia	1 (0.28)	1 (0.43)	0 (0.00)
	Caudata	Salamandra	34 (9.68)	27 (11.68)	7 (5.83)
Total	03	08	351 (99.95)	231 (99.97)	120 (99.98)
Reptiles	Reptlia	Réptil	1 (0.07)	0 (0.00)	1 (0.20)
	Squamata	Cobra	322 (22.67)	173 (18.74)	149 (29.97)
		Surucucu	9 (0.63)	8 (0.86)	1 (0.20)
		Cascavel	29 (2.04)	24 (2.60)	5 (1.00)
		Naja	8 (0.56)	6 (0.65)	2 (0.40)
		Coral	22 (1.54)	16 (1.73)	6 (1.20)
		Cipó	23 (1.61)	14 (1.51)	9 (1.81)
		Jibóia	16 (1.12)	12 (1.30)	4 (0.80)
		Corre campo	3 (0.21)	3 (0.32)	0 (0.00)
		Cobra do mato	1 (0.07)	1 (0.10)	0 (0.00)
		Cobra do sertão	1 (0.07)	1 (0.10)	0 (0.00)
		Jararaca	12 (0.84)	10 (1.08)	2 (0.40)
		Sucuri	4 (0.28)	4 (0.43)	0 (0.00)
		Lagarto	57 (4.01)	38 (4.11)	19 (3.82)
		Iguana	6 (0.42)	2 (0.21)	4 (0.80)
		Calango	5 (0.35)	0 (0.00)	5 (1.00)
		Camaleão	142 (10.00)	84 (9.10)	58 (11.67)
		Tejo	101 (7.11)	63 (6.82)	38 (7.64)
		Lagartixa	54 (3.80)	24 (2.60)	30 (6.03)
		Vibra	1 (0.07)	0 (0.00)	1 (0.20)
		Dragão	2 (0.14)	2 (0.21)	0 (0.00)
		Dragão komodo	1 (0.07)	1 (0.10)	0 (0.00)
	Crocodylia	Jacaré	221 (15.56)	160 (17.33)	61 (12.27)
		Crocodilo	89 (6.26)	76 (8.23)	13 (2.61)
	Saurischia	Disossauro	12 (0.84)	10 (1.08)	2 (0.40)
	Chelonia/Testudines	Quelônio	1 (0.07)	1 (0.10)	0 (0.00)
		Cágado	48 (3.38)	36 (3.90)	12 (2.41)
		Cágado d’água	1 (0.07)	0 (0.00)	1 (0.20)
		Jabuti	69 (4.85)	40 (4.33)	29 (5.83)
		Tartaruga	159 (11.19)	114 (12.35)	45 (9.05)
Total	05	30	1420 (99.91)	923 (99.89)	497 (99.91)
Aves	Birds	Aves	108 (18.71)	61 (25.41)	47 (13.94)
	Sphenisciformes	Pinguim	3 (0.51)	2 (0.83)	1 (0.29)
	Piciformes	Tucano	14 (2.42)	9 (3.75)	5 (1.48)
		Pica-pau	2 (0.34)	2 (0.86)	0 (0.00)
	Passeriformes	Golado***	5 (0.86)	1 (0.41)	4 (1.18)
		Concriz***	3 (0.51)	0 (0.00)	3 (0.89)
		Azulão***	7 (1.21)	3 (1.25)	4 (1.18)
		Pardal	6 (1.03)	1 (0.41)	5 (1.48)
		Sabiá	1 (0.17)	0 (0.00)	1 (0.29)
		Lavandeira	1 (0.17)	0 (0.00)	1 (0.29)
		Maria fita***	1 (0.17)	0 (0.00)	1 (0.29)
		Galo campina***	9 (1.55)	5 (2.08)	4 (1.18)
		Andorinha	1 (0.17)	0 (0.00)	1 (0.29)
		Tico-tico	1 (0.17)	1 (0.41)	0 (0.00)
		Papa capim	1 (0.17)	1 (0.41)	0 (0.00)
		Canário terra***	1 (0.17)	1 (0.41)	0 (0.00)
		Bigode***	1 (0.17)	0 (0.00)	1 (0.29)
		Lagarteiro	1 (0.17)	0 (0.00)	1 (0.29)
		Corvo	1 (0.17)	1 (0.41)	0 (0.00)
		Cardial	1 (0.17)	1 (0.41)	0 (0.00)
	Apodiformes	Beija-flor	1 (0.17)	0 (0.00)	1 (0.29)
	Psittaciformes	Papagaio	23 (3.98)	13 (5.41)	10 (2.96)
		Arara	25 (4.33)	18 (7.50)	7 (2.07)
		Arara azul	5 (0.86)	5 (2.08)	0 (0.00)
		Periquito***	11 (1.90)	6 (2.50)	5 (1.48)
		Maroca***	1 (0.17)	1 (0.41)	0 (0.00)
		Calopsita***	1 (0.17)	0 (0.00)	1 (0.29)
	Struthioniformes	Ema	14 (2.42)	1 (0.41)	13 (3.85)
		Avestruz	16 (2.77)	7 (2.91)	9 (2.67)
	Ciconiiformes	Teteu	2 (0.34)	0 (0.00)	2 (0.59)
	Falconiformes	Gavião	16 (2.77)	6 (2.50)	10 (2.96)
		Carcará	5 (0.86)	0 (0.00)	5 (1.48)
		Falcão	6 (1.03)	2 (0.83)	4 (1.18)
	Accipitriformes	Águia	8 (1.38)	3 (1.25)	5 (1.48)
		Urubu	16 (2.77)	5 (2.08)	11 (3.26)
	Columbiformes	Arribaçã	7 (1.21)	0 (0.00)	7 (2.07)
		Rolinha	16 (2.77)	1 (0.41)	15 (4.45)
		Asa branca	1 (0.17)	0 (0.00)	1 (0.29)
		Pombo***	2 (0.34)	0 (0.00)	2 (0.59)
	Tinamiformes	Lambu	2 (0.34)	0 (0.00)	2 (0.59)
	Pelecaniformes	Garça	6 (1.03)	6 (2.50)	0 (0.00)
	Apterygiformes	Kiwi	1 (0.17)	1 (0.41)	0 (0.00)
	Cariamiformes	Seriema	4 (0.69)	0 (0.00)	4 (1.18)
	Strigiformes	Coruja	6 (1.03)	5 (2.08)	1 (0.29)
	Anseriformes	Pato***	35 (6.06)	13 (5.41)	22 (6.52)
		Ganso***	3 (0.51)	0 (0.00)	3 (0.89)
	Galliformes	Guiné***	7 (1.21)	1(0.41)	6 (1.78)
		Peru***	12 (2.07)	2 (0.83)	10 (2.96)
		Galo/Galinha***	153 (26.51)	53(22.08)	100 (29.67)
		Pavão***	4 (0.69)	2 (0.83)	2 (0.59)
Total	18	50	577 (99.73)	240 (99.89)	337 (99.79)
Mammalia	Carnivorous	Leão	355 (11.17)	204 (13.71)	151 (8.93)
		Onça pintada	12 (0.37)	9 (0.60)	3 (0.17)
		Onça	179 (5.63)	96 (6.45)	83 (4.91)
		Gato***	269 (8.46)	126 (8.47)	143 (8.46)
		Cachorro***	320 (10.07)	158 (10.62)	162 (9.58)
		Tigre	140 (4.40)	90 (6.05)	50 (2.95)
		Raposa	63 (1.98)	20 (1.34)	43 (2.54)
		Leopardo	41 (1.29)	27 (1.81)	14 (0.82)
		Lobo	27 (0.84)	9 (0.60)	18 (1.06)
		Gato do mato	25 (0.78)	10 (0.67)	15 (0.88)
		Furão	1 (0.03)	1 (0.06)	0 (0.00)
		Foca	3 (0.09)	1 (0.06)	2 (0.11)
		Guepardo	12 (0.37)	8 (0.53)	4 (0.23)
		Pantera	4 (0.12)	3 (0.20)	1 (0.05)
		Urso	36 (1.13)	16 (1.07)	20 (1.18)
		Guará	2 (0.06)	0 (0.00)	2 (0.11)
		Guaxite	6 (0.18)	2 (0.13)	4 (0.23)
		Jaguatirica	7 (0.22)	1 (0.06)	6 (0.35)
		Suricato	1 (0.03)	0 (0.00)	1 (0.05)
		Puma	3 (0.09)	2 (0.13)	1 (0.05)
		Lontra	2 (0.06)	0 (0.00)	2 (0.11)
		Lince	3 (0.09)	0 (0.00)	3 (0.17)
		Coiote	1(0.03)	0 (0.00)	1 (0.05)
		Ariranha	1(0.03)	1 (0.06)	0 (0.00)
		Chacau	1(0.03)	1 (0.06)	0 (0.00)
		Jaguar	3(0.09)	0 (0.00)	3 (0.17)
		Morsa	1(0.03)	0 (0.00)	1 (0.05)
		Hiena	25 (0.78)	17 (1.14)	8 (0.47)
		Texugo do mel	1(0.03)	1 (0.06)	0 (0.00)
	Primate	Humano	1(0.03)	1 (0.06)	0 (0.00)
		Chipanzé	2 (0.06)	1 (0.06)	1 (0.05)
		Macaco	158 (4.97)	63 (4.23)	95 (5.62)
		Gorila	12 (0.37)	1 (0.06)	11 (0.65)
		Saguim	10 (0.31)	5 (0.33)	5 (0.29)
		Mico-leão-dourad	7 (0.22)	5 (0.33)	2 (0.11)
		Lêmore	1(0.03)	1 (0.06)	0 (0.00)
	Cingulata	Tatu	37 (1.16)	17 (1.14)	20 (1.18)
		Peba	6 (0.16)	2 (0.13)	4 (0.23)
	Proboscidea	Elefante	116 (3.65)	64 (4.30)	52 (3.07)
		Mamute	4 (0.12)	3 (0.20)	1 (0.05)
	Artiodactyls	Girafa	112 (3.52)	74 (4.97)	38 (2.24)
		Hipopótamo	39 (1.22)	23 (1.54)	16 (0.94)
		Boi/Vaca***	221 (6.95)	78 (5.24)	143 (8.46)
		Porco***	63 (1.98)	20 (1.34)	43 (2.54)
		Bode/cabra***	58 (1.82)	14 (0.94)	44 (2.60)
		Ovelha***	45 (1.41)	5 (0.33)	40 (2.36)
		Carneiro***	8 (0.25)	2 (0.13)	6 (0.35)
		Camelo	13 (0.40)	4 (0.26)	9 (0.53)
		Javali	18 (0.56)	14 (0.94)	4 (0.23)
		Búfalo	22 (0.69)	12 (0.80)	10 (0.59)
		Cervo	1(0.03)	1 (0.06)	0 (0.00)
		Lhama	5 (0.15)	5 (0.33)	0 (0.00)
		Gazela	1(0.03)	1 (0.06)	0 (0.00)
		Gnu	1(0.03)	0 (0.00)	1 (0.05)
		Alce	2(0.06)	0 (0.00)	2 (0.11)
	Didelphidae	Gambá	7 (0.22)	4 (0.26)	3 (0.17)
	Perissodactyla	Zebra	86 (2.70)	34 (2.28)	52 (3.07)
		Cavalo/Égua***	202 (6.35)	70 (4.70)	132 (7.81)
		Burro mulo***	6 (0.18)	3 (0.20)	3 (0.17)
		Burro/jumento***	62 (1.95)	13 (0.97)	49 (2.89)
		Rinoceronte	17 (0.53)	12 (0.80)	5 (0.29)
		Anta	13 (0.40)	8 (0.53)	5 (0.29)
		Cavalo silvestre	1(0.03)	1 (0.06)	0 (0.00)
		Burro selvagem	1(0.03)	0 (0.00)	1 (0.05)
	Lagomorpha	Lebre***	1(0.03)	1 (0.06)	0 (0.00)
		Coelho***	34 (1.07)	11 (0.73)	23 (1.36)
	Rodentia	Rato	65 (2.04)	28 (1.88)	37 (2.18)
		Porco d India***	1 (0.03)	1 (0.06)	0 (0.00)
		Hamster***	2 (0.06)	1 (0.06)	1 (0.05)
		Capivara	20 (0.62)	13 (0.97)	7 (0.41)
		Preá	11 (0.34)	1 (0.06)	10 (0.59)
		Cotia	3 (0.09)	1 (0.06)	2 (0.11)
		Paca	2 (0.06)	2 (0.13)	0 (0.00)
		Esquilo	6 (0.18)	4 (0.26)	2 (0.11)
		Porco espinho	10 (0.31)	3 (0.20)	7 (0.41)
	Cetartiodactyla	Veado	36 (1.13)	19 (1.27)	17 (1.00)
	Cetacea	Baleia	19 (0.59)	11 (0.73)	8 (0.47)
		Golfinho	2 (0.06)	1 (0.06)	1 (0.05)
		Boto	1 (0.03)	1 (0.06)	0 (0.00)
		Orca	1 (0.03)	1 (0.06)	0 (0.00)
	Diprotodontia	Canguru	8 (0.25)	6 (0.40)	2 (0.11)
		Coala	1 (0.03)	1 (0.06)	0 (0.00)
	Chiroptera	Morcego	11 (0.34)	3 (0.20)	8 (0.47)
	Didelphimorphia	Tacaca	5 (0.15)	0 (0.00)	5 (0.29)
		Timbu	8 (0.25)	2 (0.13)	6 (0.35)
	Pilosa	Tamanduá	18 (0.56)	7 (0.47)	11 (0.65)
		Preguiça	10 (0.31)	5 (0.33)	5 (0.29)
Total	15	87	3177 (99.61)	1487 (99.76)	1690 (99.57)
Invertebrates	Life stages****	Larva	1 (0.68)	0 (0.00)	1 (1.03)
	Haplotaxida	Minhoca	31 (21.08)	13 (26.00)	18 (18.55)
	Araneae	Aranha	8 (5.44)	3 (6.00)	5 (5.15)
		Caranguejeira	1 (0.68)	0 (0.00)	1 (1.03)
	Decapoda	Carangueijo	1 (0.68)	1 (2.00)	0 (0.00)
		Camarão	2 (1.36)	1 (2.00)	1 (1.03)
	Insecta*	Inseto	2 (1.36)	2 (4.00)	0 (0.00)
	Lepidoptera	Lagarta	12 (8.16)	3 (6.00)	9 (9.27)
		Borboleta	11 (7.48)	4 (8.00)	7 (7.21)
	Mollusca**	Molusco	1 (0.68)	1 (2.00)	0 (0.00)
	Scorpiones	Escorpião	1 (0.68)	1 (2.00)	0 (0.00)
	Diptera	Mosquito	2 (1.36)	0 (0.00)	2 (2.06)
		Mosca	6 (4.08)	1 (2.00)	5 (5.15)
		Muriçoca	2 (1.36)	0 (0.00)	2 (2.06)
	Hymenoptera	Formiga	10 (6.80)	3 (6.00)	7 (7.21)
		Abelha	3 (2.04)	1 (2.00)	2 (2.06)
		Maribondo	3 (2.04)	0 (0.00)	3 (3.09)
	Ascaridida	Lombriga	1 (0.68)	0 (0.00)	1 (1.03)
	Pulmonata	Lesma	3 (2.04)	0 (0.00)	3 (3.09)
		Caramujo	1 (0.68)	0 (0.00)	1 (1.03)
	Coleoptera	Besouro	5 (3.40)	2 (4.00)	3 (3.09)
	Cnidaria**	Água-viva	3 (2.04)	0 (0.00)	3 (3.09)
		Pólipo	1 (0.68)	0 (0.00)	1 (1.03)
	Siphonaptera	Pulga	1 (0.68)	0 (0.00)	1 (1.03)
	Blattodea	Barata	9 (6.12)	1 (2.00)	8 (8.24)
	Porífera**	Esponja	2 (1.36)	1 (2.00)	1 (1.03)
	Scolopendromorpha	Centopéia	1 (0.68)	0 (0.00)	1 (1.03)
	Orthoptera	Grilo	3 (2.04)	0 (0.00)	3 (3.09)
		Gafanhoto	4 (2.72)	1 (2.00)	3 (3.09)
	Phasmatodea	Mané mago	1 (0.68)	0 (0.00)	1 (1.03)
	Octopoda	Polvo	1 (0.68)	0 (0.00)	1 (1.03)
	Hemiptera	Barbeiro	1 (0.68)	0 (0.00)	1 (1.03)
	Diplopoda	Piolho de cobra	2 (1.36)	1 (2.00)	1 (1.03)
	Crustacea*	Crustáceo	1 (0.68)	0 (0.00)	1 (1.03)
Total	24	34	137 (99.96)	40 (100.00)	97 (99.92)

^a^We consider the animal denomination by the local vernacular name cited by the interviewers; *Class, **Phylum, ***Locally domestic, and ****Others

Among the socioeconomic variables verified as influential in the recognition of wild vertebrates, the data revealed a weak correlation for family income (rt = 0.06; *P* < 0.05). With regard to gender, males cited a higher number of wild, domestic, and invertebrate animals (Table 3). There was no significant difference (H = 16.79; *p* = 0.1495) with regard to religious orientation and the citation frequencies of wild vertebrates (Table 3).

**Table 3:** Socioeconomic variables and relation to animal citations frequency by the students surveyed

Categories	Variables	Wild	Domestics	Invertebrates
Gender	Female	2200 (72.0%)–122 spp.	793 (26.0%)–18 spp.	62 (2.0%)–19 spp.
	Male	2029 (71.9%)–166 spp.	718 (25.4%)–24 spp.	75 (2.7%)–34 spp.
Total		4229	1511	137
Family income*	0	209 (82.9%)	39 (15.5%)	4 (1.6%)
	1	973 (66.3%)	445 (30.3%)	49 (3.3%)
	2	2603 (72.6%)	907 (25.3%)	77 (2.1%)
	3	367 (77.4%)	102 (21.5%)	5 (1.1%)
	4	77 (79.4%)	18 (18.6%)	2 (2.1%)
Total		4229	1511	137
Religion	Not declared	801 (70.4%)	295 (25.9%)	42 (3.7%)
	Adventist	6 (85.7%)	0 (0.0%)	1 (14.3%)
	Atheist	28 (90.3%)	3 (9.7%)	0 (0.0%)
	Candomblé	7 (100.0%)	0 (0.0%)	0 (0.0%)
	Catholic	2005 (69.0%)	839 (28.9%)	61 (2.1%)
	Sciences	16 (100.0%)	0 (0.0%)	0 (0.0%)
	Christian	148 (80.9%)	32 (17.5%)	3 (1.6%)
	Spiritism	7 (100.0%)	0 (0.0%)	0 (0.0%)
	Evangelical	1125 (76.3%)	321(21.8%)	29 (2.0%)
	God’s Law	4 (100.0%)	0 (0.0%)	0 (0.0%)
	Mormom	24 (77.4%)	7 (22.6%)	0 (0.0%)
	Protestant	38 (74.5%)	12 (23.5%)	1 (2.0%)
	Jehovah’s Witnesses	20 (90.9%)	2 (9.1%)	0 (0.0%)
Total		4229	1511	137

*Income: 0 to 4—national minimum wage (based on the year 2016, US $ 232.80): 0—not declared; 1—up to 1 salary; 2—up to 2 wages; 3—up to 3 salaries; 4—more than 3

Considering the level of schooling, in general, the data revealed a positive correlation between curricular development and the citation of wild vertebrates (rs = 0.08; *P* < 0.01), while curricular development was negatively correlated with the citation of domestic vertebrates (rs = − 0.22; *P* < 0.01) and invertebrates (rs = − 0.14; *P* < 0.01) (Table 4). When analyzing the contexts separately, only the urban context presented a positive correlation between curricular development and the citation of wild vertebrates (rs = 0.09; *P* < 0.05); however, there were negative correlations between curricular development and citations of domestic vertebrates and invertebrates for, respectively, the urban (rs = − 0.11; *P* < 0.01), (rs = − 0.12; *P* < 0.01) and rural (rs = − 0.32; *P* < 0.01) (rs = − 0.16; *P* < 0.01) contexts.

**Table 4:** Curricular evolution and citation of wild, domestic and invertebrate animals by the students interviewed: means (standard deviation)

Ensino Fundamental II (elementary)	Wild	Domestic	Invertebrates
6th grade	3.5 (3.0)	2.4 (2.4)	0.2 (0.6)
7th grade	4.6 (3.5)	1.9 (2.5)	0.3 (0.9)
8th grade	5.0 (3.6)	1.3 (2.1)	0.1 (0.4)
9th grade	4.2 (3.7)	1.4 (2.3)	0.1 (0.5)
Averages	4.29 (3.48)	1.77 (2.35)	0.2 (0.7)
1st grade	4.0 (3.4)	1.4 (2.0)	0.1 (0.5)
2nd grade	4.7 (3.4)	0.5 (1.4)	0.0 (0.1)
3rd grade	5.1 (3.4)	0.9 (1.8)	0.1 (0.5)
Averages	4.43 (3.43)	1.07 (1.84)	0.1 (0.4)

In relation to age group, the general data revealed only negative correlations between age group and the citation of domestic animals (rs = − 0.11 *P* < 0.01) and invertebrates (rs = − 0.11 *P* < 0.01), an observation also made for the rural context, (rs = − 0.28 *P* < 0.01;) and (rs = − 0.13 *P* < 0.01), respectively. For the urban context, only a negative correlation was observed between age group and the citation of domestic vertebrates (rs = − 0.10; *P* < 0.01).

### Richness and diversity of the cited large groups of vertebrates

In the general context, the groups with the greatest richness of cited animals were mammals (*n* = 87; 3177 citations), birds (*n* = 50; 577 citations) and reptiles (*n* = 30; 1420 citations), while the groups with the greatest diversity were mammals (*H′* = 3.374) and birds (*H′* = 2.838) (Table 5). Values between 0.59 and 0.84% of the estimated richness per group (Table 5) were found for richness observed, with amphibians being the group with the lowest difference between observed and estimated richness (0.84). Analyzing contexts separately, mammals and birds were cited more by rural students, while fish, amphibians, and reptiles were cited more by urban students. In fact, the number of citations for reptiles by urban students (*n* = 923) was almost double that of rural students (*n* = 497) (Table 2).

**Table 5:** Observed, estimated, and diversity richness by groups of animals cited by students

Variables	Fish	Amphibians	Reptiles	Birds	Mammals	Invertebrates
Cited species richness	15	8	30	50	87	34
Number of citations	215	351	1420	577	3177	137
Diversity (*H′*)	1.32	1.29	2.499	2.838	3.374	2.94
Chao-1	25.5	9.5	40.5	74	108.4	47
Estimated richness (%)	0.59	0.84	0.74	0.68	0.80	0.72

Among the 215 citations for fish, the generic name “peixe” (fish) stood out with 66.97% of all the citations and 77.90% of the citations by rural students. The two particular citations that had the highest frequencies were “piranha” (Osteoglossiformes) in the general and urban contexts, with 8.37% and 11.62%, respectively, and “tubarão” (shark) (Selachimorpha) in the general and rural contexts, with 7.90% and 9.30%, respectively. For amphibians, there was a predominance of citations of the generic names “sapo” (toad; 48.43%), “rã” (frog; 25.35%), “perereca” (tree frog; 15.09%), and “salamandra” (salamander; 9.68%) (Table 2).

In the case of reptiles, the citation frequency for the general name “cobra” (snake) stood out in both the general (22.67%) and rural (29.97%) contexts. Citation frequencies for particular snake names were always proportionally higher among urban students (Table 2). Among lizards, in the general context, citations of “camaleão” (chameleon; 10%) and “teju” (tegu; 7.11%) were predominant. For the order Crocodylia, the citation frequencies for “jacaré” (caiman) and “crocodilo” (crocodile), stood out in the general (15.56% and 6.26%, respectively) and urban (17.33% and 8.23%, respectively) contexts. For the order Testudines, the citation frequency of generic name “tartaruga” (tortoise/turtle) was predominant in all contexts analyzed.

Among the citations for birds, the frequency of the general name “ave” (bird) stood out in the general (18.71%) and urban (25.41%) contexts. For specificities among the citations in the general context, Psittaciformes including “papagaio” (parrot; 3.98%) and “arara” (macaw; 4.33%) were emphasized; the citation frequency for the latter reached 7.50% of all the urban citations. In the order Anseriformes, the citation frequency for “pato” (duck) in the general (6.06%) and rural (6.52%) contexts stood out. Lastly, Galliformes was the most represented order for the group, with the highest citation frequency being for “galinha” (chicken) in the general (26.51%) and rural (29.67%) contexts.

For mammals, the order Carnivora was the most represented, with the domestic animals “gato” (cat) and “cachorro” (dog) being among the three most frequently cited. In addition to these, the citation frequencies for large, showy animals with media appeal stood out, including “leão” (lion), “onça” (jaguar), “tigre” (tiger), “leopardo” (leopard), “lobo” (wolf), “gato do mato” (oncilla), “guepardo” (cheetah), “urso” (bear), and “hiena” (hyena), without significant differences in frequencies between urban and rural contexts (Table 2). In the order Primates, the citation frequencies for “macaco” (monkey), “gorila” (gorilla), and “saguim” (marmoset) stood out, with the first two being cited more frequently in the rural context. The order Cingulata is highlighted by the frequency of citations for “tatu” (armadillo), an animal native to the region under study. The order Proboscidea was represented by the high frequency of citations for “elefante”, (elephant), an exotic animal.

Another order of well-represented mammals was Artiodactyla, with animals not native to the region being most frequently cited, including “girafa” (giraffe), “hipopótamo” (hippopotamus), “camelo” (camel), “javali” (boar), and “búfalo” (buffalo), among others with lower citation frequencies; as well as domestic animals traditionally used in the region and among the main sources of protein, such as “boi/vaca” (cow), which had the highest frequency of citations for the order, followed by “porco” (pig), “bode/cabra” (goat), “ovelha” (sheep), and “carneiro” (ram), which were cited much more by rural students. In the order Perissodactyla, citations for “zebra”, “rinoceronte” (rhinoceros), and “anta” (tapir) stood out, along with other domestic animals commonly raised in the studied region, such as “cavalo/égua” (horse/mare), which had the highest citation frequency for the order, and “burro/jumento” (donkey), both of which were also cited more by rural students (Table 2). The order Lagomorfa was uniquely represented by the domestic “coelho” (rabbit), which also had a higher citation frequency in the rural context.

The order Rodentia was distinguished by the citation frequencies for “rato” (rat), “capivara” (capybara), “porco da índia” (guinea pig), and “preá” (Brazilian guinea pig), the last of which is a native animal of the region and was cited mostly by rural students. The order Cetacea stood out for citations of “baleia” (whale), while for the order Chiroptera, “morcego” (bat) was emphasized, and much more so by rural students. Finally, the order Pilosa is highlighted by the citation frequencies for “tamanduá” (tamandua/anteater) and “preguiça” (sloth) (Table 2).

Despite having a relatively low citation frequency (*n* = 137), invertebrates had a proportionally high number of orders (*n* = 18), respective animal representatives (richness) (*n* = 28), and diversity (*H′* = 2.94). There was also a significant difference in the citation frequencies of the urban (29.2%) and rural (70.8%) contexts. Among invertebrate specificities, the citation frequencies of following stand out: “minhoca” (worm; Haplotaxida), more representative of the urban context; “aranha” (spider; Araneae); “lagarta” (caterpillar) and “borboleta” (butterfly) (Lepidoptera); “formiga” (ant; Hymenoptera); and barata (cockroach; Blattodea), mostly represented by the rural context.

### Origins of knowledge about the animals cited by students

**Fig. 2:**
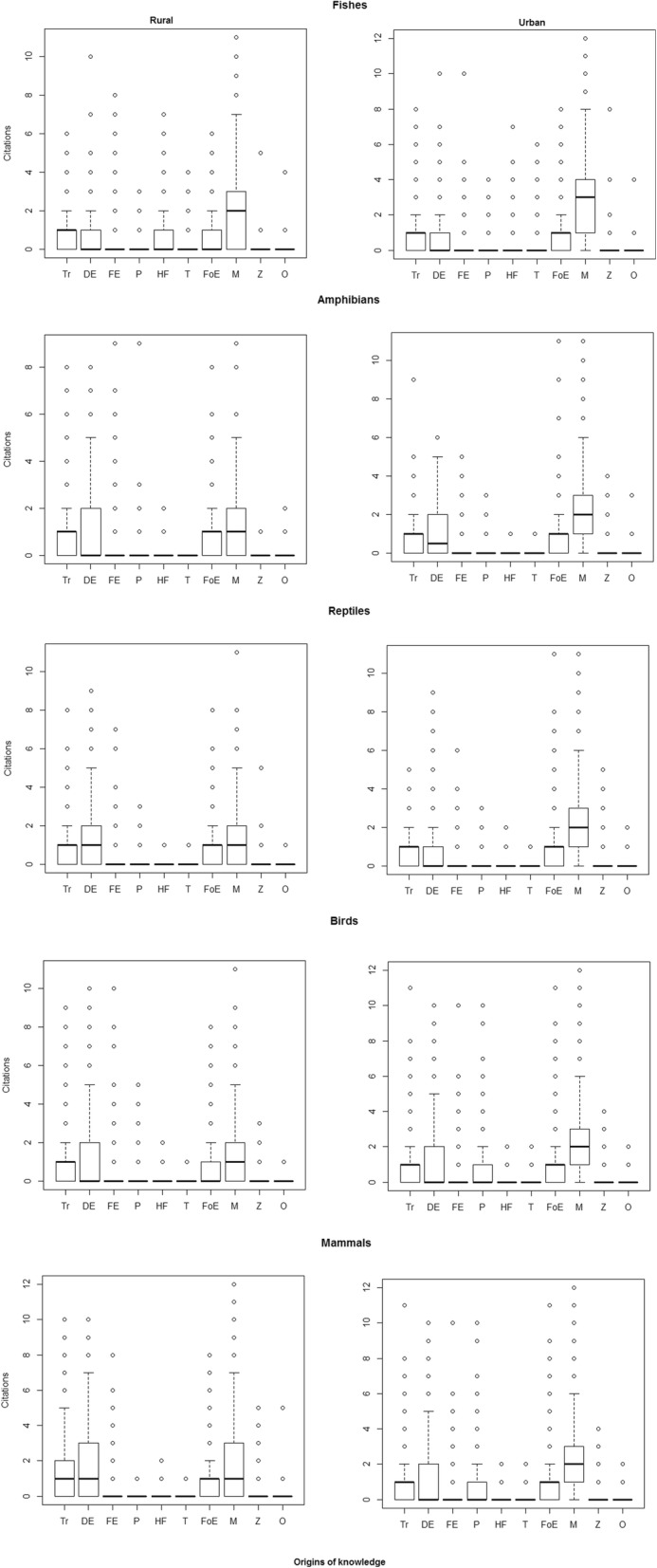
Box plot of citations from the knowledge origins by animal groups. *X*-axis acronyms—Tr, DE, FE, P, HF, T, FoE, M, Z, O—are described in Table 1

Highlighted among the categories for the origin of knowledge about the animals cited by students (Table 6; Fig. 2) are, in descending order of frequency, “media,” “daily experience,” “tradition,” and “formal education,” with the first being much more represented in terms of percentage. For all groups, the citation frequencies for “media” were always higher for urban students than for rural students; in contrast, the frequencies of citations for “tradition” in all groups, except fish, were higher for rural students. Despite having low frequencies, two other categories were noted by rural students: “field experiences” and “hunting/fishing.”

**Table 6:** Origins of the knowledge about the animals cited by the research participants

Categories	Fish (%)			Amphibians (%)			Reptiles (%)			Birds (%)			Mammals (%)		
	General	Urban	Rural	General	Urban	Rural	General	Urban	Rural	General	Urban	Rural	General	Urban	Rural
Tradition	1058 (17.3)	623 (17.7)	435 (16.7)	735 (15.5)	369 (13.6)	366 (18.1)	719 (15.0)	349 (12.7)	370 (18.1)	1113 (18.6)	568 (16.5)	545 (21.6)	1448 (21.2)	735 (19.1)	713 (23.8)
Everyday experience	774 (12.6)	439 (12.5)	335 (12.9)	1110 (23.5)	607 (22.4)	503 (24.9)	942 (19.7)	452 (16.5)	490 (24.0)	1232 (20.6)	608 (17.6)	624 (24.7)	1534 (22.4)	756 (19.7)	778 (26.0)
Field experiences	391 (6.4)	154 (4.4)	237 (9.1)	362 (7.7)	143 (5.3)	219 (10.8)	331 (6.9)	154 (5.6)	177 (8.7)	452 (7.6)	181 (5.2)	271 (10.7)	508 (7.4)	308 (8.0)	200 (6.7)
Pet	31 (0.5)	23 (0.7)	8 (0.3)	53 (1.1)	24 (0.9)	29 (1.4)	120 (2.5)	95 (3.5)	25 (1.2)	468 (7.8)	350 (10.1)	118 (4.7)	22 (0.3)	19 (0.5)	3 (0.1)
Hunting/fishing	409 (6.7)	138 (3.9)	271 (10.4)	11 (0.2)	1 (0.0)	10 (0.5)	17 (0.4)	13 (0.5)	4 (0.2)	42 (0.7)	15 (0.4)	27 (1.1)	11 (0.2)	2 (0.1)	9 (0.3)
Commerce	195 (3.2)	127 (3.6)	68 (2.6)	1 (0.0)	1 (0.0)	0 (0.0)	2 (0.0)	1 (0.0)	1 (0.0)	10 (0.2)	7 (0.2)	3 (0.1)	1 (0.0)	0 (0.0)	1 (0.0)
Formal education	672 (11.0)	408 (11.6)	264 (10.1)	850 (18.0)	521 (19.3)	329 (16.3)	807 (16.9)	498 (18.1)	309 (15.2)	727 (12.2)	442 (12.8)	285 (11.3)	933 (13.6)	525 (13.7)	408 (13.6)
Media	2532 (41.4)	1563 (44.5)	969 (37.2)	1559 (33.0)	1002 (37.1)	557 (27.5)	1726 (36.1)	1104 (40.2)	622 (30.5)	1857 (31.1)	1222 (35.4)	635 (25.1)	2238 (32.7)	1410 (36.7)	828 (27.6)
Zoological units	41 (0.7)	31 (0.9)	10 (0.4)	35 (0.7)	29 (1.1)	6 (0.3)	107 (2.2)	72 (2.6)	35 (1.7)	68 (1.1)	52 (1.5)	16 (0.6)	119 (1.7)	74 (1.9)	45 (1.5)
Others	20 (0.3)	10 (0.3)	10 (0.4)	11 (0.2)	7 (0.3)	4 (0.2)	15 (0.3)	9 (0.3)	6 (0.3)	11 (0.2)	6 (0.2)	5 (0.2)	27 (0.4)	17 (0.4)	10 (0.3)
Total	6123	3516	2607	4727	2704	2023	4786	2747	2039	5980	3451	2529	6841	3846	2995

The data revealed correlations between curricular development and the citation frequencies of the categories of the origin of knowledge about the cited animals (Table 7). For “tradition”, a significant negative correlation was observed for all groups, except fish (*P* > 0.05), in the general (amphibians rs = − 0.10; reptiles rs = − 0.11; birds rs = − 0.06; mammals rs = − 0.14; *P* < 0.01) and rural (amphibians rs = − 0.15; reptiles rs = − 0.16; birds rs = − 0.13; mammals rs = − 0.13; *P* < 0.01) contexts; in the urban area, the correlation was only for mammals (rs = − 0.15; *P* < 0.01). That is, as schooling progresses, the frequency of citations of the tradition category as the origin of knowledge about animals decreases; for the origin of knowledge about fish, the only correlation between curricular development and citing tradition was a negative one in the urban context (rs = 0.12; *P* < 0.01).

**Table 7:** Curricular evolution and citations of the four most expressive categories referring to the origins of the knowledge about the wild vertebrates by the participants: averages (standard deviation) for the Ensino Fundamental II (elementary) and Ensino Médio (upper secondary)

Categories:		Tradition			Everyday experiences			Formal education			Medias		
Group	Grade	General	Urban	Rural	General	Urban	Rural	General	Urban	Rural	General	Urban	Rural
Fish	6th	0.7 (0.5)	0.6 (0.5)	0.8 (0.5)	1.2 (1.1)	1.2 (1.2)	1.1 (1.0)	0.4 (0.5)	0.4 (0.5)	0.3 (0.4)	2.4 (2.0)	3.3 (2.3)	1.3 (1.3)
	7th	1.3 (1.0)	1.4 (1.2)	1.2 (0.8)	0.5 (0.7)	0.6 (1.0)	0.3 (0.5)	0.6 (0.7)	0.6 (0.7)	0.6 (0.7)	2.8 (1.8)	3.0 (1.9)	2.5 (1.8)
	8th	1.3 (1.0)	1.5 (1.3)	1.2 (0.9)	0.7 (1.0)	0.7 (1.0)	0.6 (0.9)	0.8 (0.7)	0.9 (0.8)	0.7 (0.6)	2.8 (1.8)	2.8 (1.6)	3.0 (2.3)
	9th	0.9 (0.6)	1.1 (0.8)	0.8 (0.5)	0.9 (1.1)	1.0 (1.2)	0.7 (0.9)	0.4 (0.6)	0.6 (0.7)	0.3 (0.4)	2.6 (2.0)	2.7 (1.9)	2.5 (2.1)
Average	EF	*1.0 (0.8)*	*1.1 (1.0)*	*1.0 (0.6)*	*0.8 (1.0)*	*0.9 (1.1)*	*0.7 (0.9)*	*0.6 (0.6)*	*0.6 (0.7)*	*0.5 (0.6)*	*2.6 (1.9)*	*3.0 (1.9)*	*2.3 (1.9)*
	1st	1.1 (0.8)	1.2 (1.0)	0.9 (0.7)	0.7 (0.9)	0.5 (0.8)	0.9 (1.1)	0.8 (0.8)	0.9 (0.7)	0.7 (0.8)	2.6 (1.9)	3.4 (2.1)	1.8 (1.4)
	2nd	1.0 (0.8)	1.1 (1.1)	0.9 (0.6)	0.8 (1.0)	0.9 (1.2)	0.7 (0.9)	1.1 (1.0)	1.4 (1.3)	0.7 (0.7)	2.0 (1.6)	2.5 (1.8)	1.4 (1.3
	3rd	1.5 (1.3)	2.1 (1.6)	1.0 (0.6)	0.9 (1.2)	0.9 (1.2)	1.0 (1.1)	1.1 (0.8)	1.2 (0.8)	1.0 (0.8)	2.2 (1.6)	2.4 (1.7)	2.0 (1.6)
Average	EM	*1.1 (0.9)*	*1.3 (1.1)*	*0.9 (0.7)*	*0.8 (1.0)*	*0.7 (1.0)*	*0.8 (1.1)*	*0.9 (0.8)*	*1.1 (0.9)*	*0.8 (0.8)*	*2.4 (1.8)*	*3.0 (2.0)*	*1.7 (1.5)*
Amphibian**s**	6th	0.6 (0.5)	0.6 (0.5)	0.7 (0.5)	1.2 (1.2)	1.5 (1.3)	0.9 (1.0)	0.5 (0.6)	0.6 (0.6)	0.4 (0.5)	1.5 (1.2)	1.9 (1.3)	0.9 (0.7)
	7th	1.0 (0.8)	0.7 (0.7)	1.2 (1.0)	1.1 (1.2)	1.0 (1.1)	1.1 (1.3)	0.8 (0.6)	0.8 (0.7)	0.7 (0.6)	1.5 (1.2)	1.7 (1.4)	1.3 (1.0)
	8th	1.0 (0.9)	0.9 (0.8)	1.2 (1.0)	0.8 (1.1)	0.9 (1.1)	0.8 (1.0)	1.1 (0.9)	1.3 (1.0)	0.9 (0.7)	2.0 (1.4)	2.4 (1.6)	1.3 (1.1)
	9th	0.7 (0.7)	0.8 (0.8)	0.6 (0.6)	1.3 (1.3)	1.4 (1.3)	1.3 (1.3)	0.7 (0.7)	0.7 (0.8)	0.7 (0.7)	1.6 (1.2)	1.6 (1.1)	1.5 (1.3)
Averages	EF	*0.8 (0.7)*	*0.7 (0.7)*	*0.9 (0.7)*	*1.1 (1.2)*	*1.2 (1.2)*	*1.0 (1.2)*	*0.8 (0.7)*	*0.9 (0.8)*	*0.7 (0.7)*	*1.6 (1.3)*	*1.9 (1.4)*	*1.3 (1.0)*
	1st	0.7 (0.6)	0.7 (0.6)	0.6 (0.6)	1.1 (1.1)	1.0 (1.2)	1.1 (1.1)	1.0 (0.7)	1.1 (0.8)	0.8 (0.7)	1.5 (1.3)	1.7 (1.3)	1.3 (1.2)
	2nd	0.4 (0.5)	0.4 (0.5)	0.3 (0.5)	1.0 (1.0)	0.8 (1.0)	1.2 (1.2)	1.2 (0.9)	1.6 (1.3)	0.8 (0.5)	1.4 (1.2)	1.9 (1.4)	0.8 (0.7)
	3rd	0.6 (0.5)	0.4 (0.5)	0.7 (0.6)	1.4 (1.2)	1.0 (1.2)	1.5 (1.2)	1.1 (0.6)	1.2 (0.7)	1.0 (0.5)	1.4 (1.1)	1.6 (1.1)	1.0 (0.9)
Averages	EM	*0.6 (0.6)*	*0.6 (0.6)*	*0.5 (0.6)*	*1.1 (1.1)*	*1.0 (1.1)*	*1.2 (1.2)*	*1.1 (0.7)*	*1.3 (0.9)*	*0.8 (0.6)*	*1.5 (1.2)*	*1.8 (1.3)*	*1.1 (1.0)*
Reptiles	6th	0.6 (0.5)	0.6 (0.5)	0.7 (0.5)	0.9 (1.0)	0.9 (1.0)	0.8 (0.9)	0.4 (0.5)	0.5 (0.6)	0.3 (0.4)	1.5 (1.1)	1.8 (1.3)	1.0 (0.6)
	7th	1.0 (0.8)	0.6 (0.7)	1.3 (1.1)	0.9 (1.0)	0.8 (1.0)	1.0 (1.0)	0.8 (0.7)	0.8 (0.7)	0.7 (0.6)	1.7 (1.3)	2.0 (1.5)	1.5 (1.1)
	8th	1.0 (0.8)	0.9 (0.7)	1.2 (1.0)	0.7 (0.9)	0.7 (0.8)	0.7 (1.0)	1.0 (0.7)	1.2 (0.8)	0.8 (0.6)	1.9 (1.2)	1.9 (1.1)	1.7 (1.3)
	9th	0.6 (0.6)	0.6 (0.6)	0.5 (0.6)	1.2 (1.2)	1.3 (1.3)	1.2 (1.1)	0.7 (0.7)	0.7 (0.7)	0.7 (0.7)	1.9 (1.3)	2.2 (1.4)	1.5 (1.3)
Averages	EF	*0.8 (0.7)*	*0.7 (0.6)*	*0.9 (0.8)*	*0.9 (1.0)*	*0.9 (1.0)*	*0.9 (1.0)*	*0.7 (0.7)*	*0.8 (0.7)*	*0.6 (0.6)*	*1.7 (1.2)*	*2.0 (1.4)*	*1.4 (1.1)*
	1°	0.7 (0.6)	0.8 (0.6)	0.6 (0.6)	1.0 (1.0)	0.7 (0.8)	1.2 (1.3)	0.8 (0.7)	1.0 (0.6)	0.6 (0.6)	1.9 (1.4)	2.3 (1.6)	1.4 (1.1)
	2°	0.4 (0.5)	0.4 (0.5)	0.3 (0.5)	1.0 (1.1)	0.7 (1.0)	1.3 (1.2)	1.3 (1.0)	1.7 (1.4)	0.8 (0.6)	1.7 (1.4)	2.2 (1.7)	1.2 (1.0)
	3°	0.6 (0.6)	0.4 (0.6)	0.7 (0.6)	1.3 (1.3)	0.8 (1.2)	1.6 (1.3)	1.3 (0.9)	1.3 (1.1)	1.0 (0.7)	1.7 (1.5)	2.0 (1.4)	0.9 (0.8)
Averages	EM	*0.6 (0.6)*	*0.6 (0.6)*	*0.5 (0.6)*	*1.0 (1.1)*	1.0 *(1.0)*	*1.3 (1.3)*	*1.0 (0.7)*	*1.3 (1.0)*	*0.8 (0.6)*	*1.8 (1.4)*	*2.3 (1.6)*	*1.2 (1.1)*
Birds	6°	0.8 (0.5)	0.8 (0.4)	0.9 (0.6)	1.0 (1.1)	1.1 (1.1)	0.9 (1.0)	0.4 (0.5)	0.5 (0.5)	0.4 (0.5)	1.5 (1.3)	2.1 (1.4)	0.9 (0.8)
	7°	1.4 (1.2)	1.1 (0.9)	1.8 (1.3)	1.0 (1.2)	1.2 (1.3)	0.7 (1.1)	0.6 (0.6)	0.6 (0.6)	0.5 (0.6)	1.8 (1.5)	2.1 (1.7)	1.6 (1.3)
	8°	1.6 (1.2)	1.5 (1.1)	1.7 (1.2)	0.9 (1.0)	0.9 (1.1)	0.9 (1.0)	1.0 (0.8)	1.1 (0.9)	0.8 (0.7)	2.0 (1.3)	2.2 (1.2)	1.7 (1.5)
	9°	0.9 (0.7)	1.0 (0.8)	0.8 (0.6)	1.5 (1.6)	1.3 (1.4)	1.8 (1.9)	0.6 (0.7)	0.7 (0.8)	0.5 (0.6)	1.9 (1.5)	2.3 (1.5)	1.4 (1.4)
Averages	EF	*1.2 (0.9)*	*1.1 (0.7)*	*1.3 (1.0)*	*1.1 (1.2)*	*1.1 (1.2)*	*1.1 (1.3)*	*0.6 (0.7)*	*0.7 (0.7)*	*0.5 (0.6)*	*1.8 (1.4)*	*2.2 (1.4)*	*1.4 (1.3)*
	1°	1.1 (0.8)	1.2 (0.9)	0.9 (0.7)	1.7 (1.7)	1.5 (1.4)	2.0 (1.9)	0.8 (0.7)	0.9 (0.7)	0.6 (0.7)	2.0 (1.6)	2.6 (1.9)	1.4 (1.3)
	2°	0.8 (0.8)	0.8 (0.8)	0.8 (0.7)	1.0 (1.1)	0.7 (0.8)	1.5 (1.5)	1.2 (1.2)	1.5 (1.4)	1.0 (0.9)	2.4 (2.1)	3.2 (2.5)	1.6 (1.4)
	3°	1.1 (0.9)	0.9 (1.1)	1.1 (0.8)	2.0 (1.8)	1.2 (1.7)	2.5 (2.0)	1.1 (1.0)	0.9 (1.0)	1.2 (1.0)	1.7 (1.4)	2.2 (1.7)	1.2 (1.0)
Averages	EM	*1.0 (0.8)*	*1.1 (0.9)*	*0.9 (0.7)*	*1.6 (1.6)*	*1.3 (1.4)*	*1.9 (1.9)*	*1.0 (0.8)*	*1.1 (0.9)*	*0.8 (0.8)*	*2.1 (1.7)*	*2.7 (2.1)*	*1.4 (1.3)*
Mammals	6°	1.0 (0.6)	1.0 (0.5)	1.1 (0.8)	1.3 (1.2)	1.6 (1.3)	1.0 (1.1)	0.5 (0.6)	0.6 (0.6)	0.4 (0.5)	1.7 (1.5)	2.3 (1.8)	0.9 (0.9)
	7°	2.6 (2.0)	2.4 (1.7)	2.7 (2.3)	0.9 (1.2)	1.2 (1.4)	0.7 (1.0)	0.9 (0.6)	1.0 (0.5)	0.9 (0.7)	2.3 (1.9)	3.2 (2.1)	1.6 (1.4)
	8°	1.9 (1.4)	1.7 (1.3)	2.3 (1.8)	1.0 (1.2)	0.8 (1.0)	1.3 (1.7)	1.1 (0.7)	1.2 (0.9)	1.0 (0.4)	2.8 (1.9)	2.9 (1.8)	2.6 (2.0)
	9°	1.3 (1.0)	1.5 (1.2)	1.1 (0.6)	2.1 (1.8)	1.6 (1.5)	2.6 (2.2)	0.8 (0.8)	1.0 (0.9)	0.6 (0.6)	2.2 (1.9)	2.4 (1.8)	2.0 (1.9)
Averages	EF	*1.7 (1.4)*	*1.6 (1.2)*	*1.8 (1.6)*	*1.3 (1.4)*	*1.3 (1.3)*	*1.4 (1.6)*	*0.8 (0.7)*	*0.9 (0.7)*	*0.7 (0.6)*	*2.2 (1.8)*	*2.7 (1.9)*	*1.7 (1.6)*
	1°	1.0 (0.8)	1.0 (0.8)	1.1 (0.8)	2.1 (1.9)	1.7 (1.5)	2.5 (2.3)	1.1 (0.9)	1.1 (0.8)	1.1 (0.9)	2.2 (2.0)	2.7 (2.3)	1.7 (1.6)
	2°	0.8 (0.8)	0.9 (0.8)	0.8 (0.8)	1.5 (1.4)	1.3 (1.2)	1.7 (1.5)	1.4 (1.1)	1.5 (1.3)	1.3 (0.9)	2.5 (1.9)	3.0 (2.2)	1.9 (1.4)
	3°	1.0 (0.8)	0.7 (0.7)	1.0 (1.0)	2.7 (1.9)	2.0 (1.7)	2.9 (2.0)	1.3 (1.0)	1.0 (0.8)	1.6 (1.4)	2.3 (1.7)	2.2 (1.8)	2.6 (1.5)
Averages	EM	*1.0 (0.8)*	*0.9 (0.8)*	*1.0 (0.8)*	*2.0 (1.7)*	*1.7 (1.6)*	*2.4 (2.1)*	*1.2 (1.0)*	*1.2 (1.0)*	*1.3 (1.0)*	*2.3 (1.9)*	*2.6 (2.2)*	*2.0 (1.5)*

*EF* Ensino Fundamental II (elementary); *EM* Ensino Médio (upper secondary)

For the “daily experience” category, the data showed a significant positive correlation for all groups, except for amphibians (*P* > 0.05), in the general context (fish rs = 0.07; reptiles rs = 0.07; birds rs = 0.14; mammals rs = 0.18; *P* < 0.05); this was also true in the rural context (amphibians rs = 0.12; reptiles rs = 0.13; birds rs = 0.26; mammals rs = 0.30; *P* < 0.01), with the exception of fish (*P* > 0.05). That is, curricular development coincides with increasing citation frequency for this category; in the urban context, only a negative correlation was observed in relation to fish (rs = − 0.11; *P* < 0.01).

Regarding “formal education,” the data revealed a significant positive correlation with curricular development for all groups analyzed in the contexts: general (fish rs = 0.15; amphibians rs = 0.19; reptiles rs = 0.20; birds rs = 0.13; mammals rs = 0.16; *P* < 0.01), rural (fish rs = 0.12; amphibians rs = 0.19; reptiles rs = 0.19; birds rs = 0.14; mammals rs = 0.24; *P* < 0.01), and urban (fish rs = 0.18; amphibians rs = 0.20; reptiles rs = 0.21; birds rs = 0.13; mammals rs = 0.09; *P* < 0.01). That is, with the progressive implementation of schooling, the frequency of citation of this category increases as a source of knowledge about animals. For “media,” the data showed a significant positive correlation only for mammals in the general (rs = 0.06; *P* < 0.05) and rural (rs = 0.19; *P* < 0.01) contexts.

## Discussion

### Understanding of wild vertebrates among urban and rural students: cited animals and influences of socioeconomic variables

The citation of domestic animals (25.7%), as well as invertebrates (2.3%), in the representation of what is understood as wild vertebrates is a situation that evidences the need for adjustments to the educational processes that address the subject. According to Brazilian curricular guidelines for basic education, in addition to other theoretical orientations [5, 6, 9], it is expected that students, beginning in the initial grades of schooling, will be able to identify animals as wild or domestic and as vertebrates or invertebrates. In other words, the ability to identify and/or “name” animals constitute the most basic level of knowledge, as well as a fundamental component for the understanding and “protection” of the diversity of life [22]. Corroborating these arguments, in a study carried out in Turkey, Yorek [1] emphasized as a priority student conceptual understanding of biological diversity for its conservation, beginning with “primary schooling,” with a focus on the relevance of revising of all aspects of the curriculum. From this perspective, among the main components guiding “environmental literacy” is conceptual knowledge, which is addressed in the early stages of early childhood education, and consequently affects the perception of issues of nature conservation and, therefore, the success of conservation initiatives [11], in addition to being fundamental to subsequent learning [4].

Our study found that the citation frequency for domestic animals and invertebrates was higher in the rural context, suggesting that if the curricular orientation is the same in both rural and urban contexts, the material conditions and forms of rural schooling approaches may be less efficient at transmitting knowledge about fauna. A similar situation has also been observed in previous studies, such as the research developed by Pinheiro et al. [13], which analyzed children’s perceptions of snakes, and concluded that access to the media, as well as the level of formal education, for rural students is generally less efficient than for urban students. In a study with Colombian indigenous communities, Páramo and Galvis [7] also observed that children do not differentiate domestic and wild animals.

Income of the studied students was found to have no influence on their knowledge about vertebrates, which diverges from the tendency observed in other studies. According to Campos et al. [3] and Rosalino et al. [11], in the contemporary context, access to the media (which presupposes purchasing power) potentializes educational processes related to human/nature relationships. Another factor that had no influence on the citation of wild vertebrates by the research participants was religious orientation, which also differs from the results of other studies, which pointed to religiosity as one of the sociocultural factors that influence the perception of animals [13, 16, 26].

On the other hand, gender was found to influence the recognition of vertebrates, with a greater richness and variety of wild, domestic, and invertebrate animals being cited by male students, suggesting that they possess more interest and knowledge about the fauna than do females. These results corroborate those obtained by other studies, which indicate that males have more affinity and knowledge about animals than females [2, 3, 7, 13, 16, 17, 29, 30, 32]. Among the possible reasons for this finding, authors suggest that males experience more pressure from parents and colleagues in the sense of encouragement towards animals, which is not observed in relation to females; in addition, females are less likely to explore the environment, and thus have contact with animals [7, 22, 32]. These explanations can also be expanded to gender differences, suggesting hormonal, genetic, evolutionary, and sociocultural factors, among others [3, 13, 26, 34, 42].

The positive correlation between curricular development and the citation of wild vertebrates observed in the general and urban contexts, as well as the negative correlations for citations of domestic vertebrates and invertebrates in the general, urban, and rural contexts, is not surprising and is possibly a consequence of the cumulative effect of a curricular approach in which biological contents are treated repeatedly, in depth, as cycles of basic education are completed, as foreseen in the national curricular guidelines [5, 6, 33], following a curricular logic of cognitive tendency [3, 9, 43–46]. In other words, despite the criticism of the current “rationalist academic, reproductive curriculum” approach, emphasized by several authors [9, 47–53], it is necessary to consider its cumulative effect on the consequences of learning. Previous studies have also identified influences of schooling on human relations with nature. For example, research developed by Pinheiro et al. [13] on children’s perceptions of snakes in the semi-arid region of Brazil, concluded that the higher level of schooling the lower the frequency of negative perceptions of these animals. Similarly, in a study carried out in South Africa, Tarrant et al. [20] recorded variation in cultural beliefs about frogs as a function of the educational levels of the respondents; that is, the less educated, the stronger the myths about these animals. These studies confirm the correlation between a person’s level of education and pro-conservationist attitudes [11]. However, it should be noted that other studies did not find any influence of level of schooling on the perception and recognition of animals [1, 17], showing the complexity of very specific phenomena of sociocultural nature, given the multiplicity of variables involved, thus making it difficult to come to conclusions and/or generalizations.

When we consider the rural context alone, the non-occurrence of a positive correlation between schooling and citation of wild vertebrates among the students surveyed may be related to the greater possibility of student contact with animals in rural environments regardless of the education level. This situation has been observed in previous studies [11, 25, 32]. From this perspective, studies show that ecological knowledge regarding the naming of species and their use is related to the level of resource dependence and frequency of environmental interaction, and thus people from rural communities hold more in ecological knowledge about animals [3]. Moreover, in the present study, this result may be indicative of little influence by the curricular approach practiced, being not based on contextualization with a focus on the local fauna. In convergence with this conclusion, Páramo and Galvis [7] emphasize that learning about fauna in rural and urban schools seems to be dissociated from direct experiences with animals and that the images and data in the texts used do not arouse interest in children because they do not portray animals of their daily reality. Finally, Pinheiro et al. [13] conclude that the level of formal education and access to information for rural students are generally lower than that for urban students.

The negative correlations between age and citation of domestic animals and invertebrates observed among the students interviewed in the present study indicate a more coherent understanding of the denomination “wild vertebrates” with increasing age, leading us to conclude that it is a consequence of the process of individual development itself, permeated by diverse cultural influences, such as media and educational processes, in general inherent to the contemporary context. This inference finds support in previous studies, which have evidenced influences of age on knowledge and perception about the diversity of life [2, 4, 15, 19, 20, 28], including, it is suggested, media and books as sources of knowledge [3, 16].

### Richness and diversity of animals cited by students

The higher richness of mammal, birds and reptile species, as well as the greater diversity among mammals and birds, is consistent with results found in previous studies [2, 8, 15, 19, 22], which indicate a trend for greater human affinity with mammals and birds. This situation may be influenced by phylogenetic proximity and thus the greater coexistence with representatives of these groups in the course of human evolution, for various purposes, such as pets or nutritional resources, as well as issues related to esthetics, behavior, and vocalization, among others. All of these factors, according to Zhang et al. [32], are convergent with the development of human interest and affection for animals. From this perspective, a study developed by Campos et al. [3] on the familiarity of urban and rural children with animals in the arid region of Argentina, found that almost 70% of the recognized animals were mammals. In the case of the richness of reptile citations observed in the present study, we suggest that local/regional traditional influences of histories and myths related to conflicts between humans and some representatives of this group, such as snakes, contribute to the insertion of these animals into the collective imagination, as well as the use of others, such as lizards and testudines, as nutritional sources and pets. These inferences are in line with findings from other studies [1, 7, 13, 17]. In summary, several studies suggest that animal perceptions result from evolutionary, cultural, and/or individual pressures, including esthetic, utilitarian, conflictual, and interactive dimensions, among others; species phylogenetically closer to humans are preferred over those phylogenetically more distant [3, 16, 20, 32, 34, 54].

Among mammals, the order Carnivora was the most represented, especially those that are large and showy, including exotic as well as domestic animals (cat and dog), evidencing a strong utilitarian role with a strong affective appeal. Mammals have been found to stand out among the animals recognized by humans in different situations [3, 7, 16–18, 32], being influenced by, among other factors, the phylogenetic proximity of these animals to humans, and thus a history of friendly utilitarian and conflictual relationships, as well as esthetic and media influences. Not surprisingly, therefore, in our study, there were high citation frequencies for animals such as “macaco” (monkey), “gorila” (gorilla), “saguim” (marmosets) (Primates), and “elefante” (elephant) (Proboscidea). Animals such as “tatu” (armadillo) (Cingulata) and “tamanduá” (tamandua/anteater) (Pilosa), native to the study region, and common hunting targets, potentiate direct interactions with them and their exploitation. This conclusion is supported by previous studies [17, 20, 31]. This situation is reinforced by the fact that wild animals native to the region or raised as domestic animals were much more represented by rural students, reflecting a greater interaction with these animals, given the specificities of the context itself, as well as the tradition of their local use as important sources of protein, highlighting their utilitarian bias. These conclusions converge with the results of other studies [2, 3, 7, 13, 30, 31]. In addition, this reinforces the idea that human preferences for species within each animal group vary [16, 31] and are motivated by factors other than utility, such as esthetics and/or morphological appearance and behavior [8, 22, 24].

The high frequency of citations observed for “coelho” (rabbit) (Lagomorpha) suggests that, in addition to the utilitarian and esthetic aspects already mentioned for other orders of mammals, the historical symbolism of religious and playful characters related to the animal and which strongly appeal to in the media, such as in commercial situations of the Christian religious calendar (Easter) and use in illusionist practices (use by magicians), among others, is important. In these cases, “symbolism” refers to the use of nature for metaphorical expressions through language [7]. A study developed by Knight [31] in the USA highlights, among others, the pygmy rabbit as being classified by students as a more esthetically pleasing species.

In the order Rodentia, the citation frequencies detected lead us to very peculiar conclusions. In the case of “rato” (rat), its presence in the imagination of students can be explained by the fact that this animal presents itself historically as an “intruder” in the environment of the human home and thus is stigmatized as a noxious animal. Other studies also emphasize the influence of the notion of animal harm to humans [3, 16–18]. On the other hand, rodents like the “preá” (Brazilian guinea pig), much cited by the rural students of the present study, are a common hunting target in the region. In this case, the utilitarian aspect of animals for humans is reinforced, which has also been evidenced in other studies [1, 7, 13, 31]. The role of the media can explain the high citation frequencies for “capivara” (capybara) and “porco da índia” (guinea pig), since they are animals not commonly found in the environment of the study. A similar situation applies to mammals such as “veado” (deer) (Cetartiodactyla), “baleia” (whale) (cetácea), and “preguiça” (sloth) (Pilosa). Studies suggest that attitudes and interests directed towards these animals by humans reflect direct interactions and experiences with them, yet are also influenced by a variety of media influences [7, 17, 32].

Some cases, such as the high citation frequency for “morcego” (bat) (Chiroptera), which was more strongly expressed by rural students, may reflect influences of stories, legends, and myths, almost always with a negative connotation, in their relations with humans, provided by diverse media sources, such as cinematography. These conclusions are supported by other studies [2, 12, 15, 31, 55]. In addition, there is a greater possibility of contact with these animals for rural students, thus reiterating the role played by direct and media experiences in determining interest in animals [17, 30].

In other vertebrate groups, the prevalence of citations for generic names such as “ave” (bird), “cobra” (snake) (Squamata), “sapo” (toad), “rã” (frog), “perereca” (tree frog) (Anura), “salamandra” (salamander) (Caudata), and “peixe” (fish), suggests a limitation in the knowledge about the diversity of these groups. This may reflect a lack of interest in these animals that is linked to the idea of “noxiousness,” little utility to humans, or other morphological or behavioral aspects, among others. Furthermore, little attention is given to these groups in educational processes. According to Tarrant et al. [20], studies with students in South Africa have identified that conceptual limitations regarding amphibians is common, even among educators.

Among the birds mentioned, the citation frequencies for “papagaio” (parrot) and “arara” (macaw) (Psittaciformes) stood out, with the latter being cited more in the urban context. This finding leads us to infer that, as supported by previous studies, influences of aspects such as showy appearance and behavior, in addition to local traditions of keeping these animals as pets, are involved [56–58]. Domestic birds, such as “pato” (duck) (Anseriformes) and “galinha” (chicken) (Galliformes), also had high citation frequencies, which reflect their importance as a protein source, especially regarding the latter. Thus, the suggested categorizations of the relationships between humans and nature, among which are esthetics and/or appearance and animal utilitarianism by humans, are reinforced [1, 7, 8, 13, 27, 31].

Snakes were the most frequently cited animals among reptiles, and especially among urban students, reflecting, among other possible factors, the role played by the media, which is more accessible in that context. A similar justification applies to other reptiles, such as “jacaré” (caiman) and “crocodilo” (crocodile) (Crocodylia), as well as the citation frequencies for fish, such as “piranha” (Osteoglossiformes) and “tubarão” (shark) (Selachimorpha), observed in our study. In these cases, the influence of the media in animal recognition is reiterated, as emphasized in previous studies [3, 13, 17, 22, 25, 26, 30]. As for lizards, the high representation of “camaleão” (chameleon) and “teju” (tegu), among the studied students is probably due to the fact that they are popular animals in the region given that they are targets of hunting activity [59, 60]. In this sense, the importance of direct experiences and utilitarian bias in reinforcing attitudes and interests regarding animals is emphasized [7, 18, 32]. Lastly, the frequencies of citations for “tartaruga” (tortoise/turtle) (Testudines), which likely refers to terrestrial “jabutis” (tortoises), also reflects their popularity as pets in the region of Brazil [60].

The high citation frequencies for exotic animals reverberates the importance of the three-dimensional complexity of ethnobiological approaches—corpus, cosmos, and praxis [61–63]. In this context, the media can insert content into the imagination, which, consequently, results in re-signification of the cosmological dimension, thus influencing the praxis of human interactions with nature. That is, in contemporary societies, much of what is known and expressed results from a much larger symbolic-virtual dimension, given the strength of the presence of information in a context of technological globalization. Thus, much of what is expressed about animals does not necessarily result from direct experiences, but from other forms of interaction [18]. It is suggested, therefore, that transition and/or hybridization occurs with respect to the nourishing elements of the cosmological dimension—from myths, legends, and beliefs in the pre-technological contexts—to diverse media contents in the contexts of technological globalization, not necessarily in a manner of substitution, but as a possibility of amplification and/or re-signification of cosmological dimension. The importance of the dimension of media to knowledge about fauna has been observed in several studies [3, 17, 22, 26, 32].

The diversity of invertebrates cited in the present study is mostly due to their greater frequency of citation by rural students, which may be indicative of a limited understanding of the vertebrate vs. invertebrate distinction. The most frequently cited invertebrates were those that are most present in human daily life, especially in the rural context. Our results converge with a study developed by Campos et al. [3] in Província de Mendonza, Argentina, in which children from urban and rural schools named 33 invertebrate species, which were most representative in the rural environment and corresponded to the third highest frequency among the animal groups cited. However, other studies emphasize that because there is a human tendency for negative reactions towards invertebrates, such as fear, antipathy, and aversion, compared to other animal groups, they would be at a “disadvantage” in the human imagination and, therefore, less often remembered and cited [1, 16, 31].

### Origins of knowledge about the animals cited

The greater citation frequency observed for “media” as the origin of knowledge about animals, especially by urban students, reflects the strength of contemporary technological globalization. Access to information on the diversity of life through technological resources, such as media tools, has been emphasized by several studies [7, 11, 22, 30, 32]. This may explain, inclusively, the high citation frequencies for exotic animals, as well as other animals that are unlikely to be involved in direct experiences with the students investigated here. A similar situation was also observed in previous studies. A study developed with students in the semi-arid region of Brazil, for example, attributes to the media the recognition of exotic species of snakes by local students [17]. However, in spite of expanding the possibilities for exploration of the natural world, this source of information can also be problematic, considering the following reflection: To what extent is media based on educational priorities? In consonance with this question, the Brazilian curricular guidelines for basic education indicate that the simple propagation of environmental problems in the media that has been observed in the last few decades does not ensure the acquisition of information and concepts endorsed by the sciences and frequently trivializes scientific knowledge [5, 6, 33]. In addition, the potentialization of knowledge about exotic animals, despite its importance, does not impart meaning to the development of critical awareness towards animal conservation in the complex local-global dimension if not done concomitantly with the recognition of local fauna. This situation points to a trend recorded by other studies, which indicated greater recognition of exotic animals in the contexts studied [3, 7]. This is an issue that raises the importance of contextualization in the processes of biological education regarding fauna and its conservation [6, 7, 30, 33, 64].

The positive correlation observed between curricular development and the citation of “media” as the origin of knowledge for only mammals in the general and rural contexts, suggests that there is little interaction between these two instances of knowledge and information. In our view, these should be brought together by incorporating media into the processes of contemporary education, especially in biological literacy, given its relevance, attractiveness, and/or technological influence. This reasoning is in consonance with other studies [3, 9–11].

“Daily experiences” is the category that brings together the second set of indications most frequently cited for the origin of knowledge about animals, and reflects the importance of daily interactions with fauna, especially in direct situations. Several studies have emphasized the importance of direct experience in human relations with animals for contributing to, among other acquisitions, demystification and critical awareness for animal conservation [7, 10, 11, 17, 18, 31, 32]. The positive correlation observed between citing of this category for the origin of knowledge about animals and curricular development can be understood, we conclude, as an expected consequence because “daily experiences” are continuous processes throughout life and thus are nourished by the confluence of the factors of schooling. In this sense, more diverse and direct experiences with animals, including field activities, among others, potentialize the possibilities of knowledge about nature [7, 10, 11, 13, 19, 20, 32].

In sequence, the data reveal that the category “tradition,” as a source of knowledge of animals, is more frequently cited by rural students, reflecting the importance of diverse cultural aspects transmitted mainly by parental interactions and between pairs. The acquisition of knowledge via these circumstances has been emphasized by previous studies [11, 24, 65, 66] suggesting that children learn about animals from a wide variety of cultures, including direct experiences with their peers and parents. In this context, studies have exemplified myths related to snakes being transmitted between generations through oral tradition [13, 17, 30, 64, 67, 68]. The negative correlation observed between curricular development and the citation of this category as a source of knowledge about animals allows us to infer that it is a consequence of the influence of intellectual development itself, in which processes of schooling participate by increasing the references of origins of knowledge about wildlife. That is, formal education should contribute to the reconstruction and expansion of knowledge about nature [13, 69].

Finally, the data place “formal education” among the categories that were most frequently cited as origins of knowledge about the fauna by the studied students, converging with the results of previous studies that emphasized the role of schooling in the processes of knowledge acquisition regarding animals and their conservation [3, 7, 10, 11, 13, 17]. However, schooling is not the main source of knowledge about fauna compared to the other categories recorded in our study, which raises some important reflections. In modern societies, formal education should figure as the first and/or central reference in the generation of knowledge. Given the fact that the data of this research were generated in the formal education context itself—Science/Biology classes—it would be expected that this circumstance would exert a greater influence in the indication of sources of knowledge about animals by the students. In addition, according to national curricular guidelines [5, 6, 33], approaches to biological content must prioritize processes of contextualization by incorporating everyday knowledge, traditions, informal sources (e.g., media), and above all, modern technological resources, in a perspective of knowledge reconstruction with a view to the development of critical consciousness of the relationships between humans and nature. From this perspective, Krasilchik [52] points out that curricular experience in contemporary schools reflects a disagreement between theory and practice; that is, a practice that is much more “academic-rationalist, fragmentary, banking” [9, 47, 48, 50–53], and much less contextual, problematizing, meaningful, progressive [6, 9, 47, 51–53, 69–72], and technologically current.

As for the positive correlation observed between the citation of this category (formal education) for the origin of knowledge about animals and curricular development, we understand it to be a consequence of the cumulative effect of the schooling process itself. According to the curricular orientation practiced, biological contents are repeatedly approached in a continually deeper manner with the advancement through the complement of school grades [5, 6, 33].

Two other categories have high citation frequencies by only rural students: “field experiences” and “hunting/fishing.” We suggest, supported by Rosalino et al. [11], that these categories are a consequence of specific contextual specificities and lifestyle. A study developed by Campos et al. [3] in a region of Argentina, related the greater familiarity of rural students with birds to the practice of hunting certain species of the local avifauna for commercial purposes, despite its illegality. Thus, the importance of direct experiences, such as recreational activities, for the development of naturalistic attitudes [10, 31, 32, 67] contributes to the recognition of fauna.

Finally, we highlight that although separated by didactic questions and data fidelity, the categories for the origins of knowledge about the fauna defined in this study are not mutually exclusive. On the contrary, in the practice of life, they converge and complement each other. That is, in contemporary reality, what is expressed by a given phenomenon reflects a simultaneous confluence of determinants that hybridize and/or complement each other, making it difficult to specify, in the field of practical experience, what configured a given expression, thus influencing formal, informal, and non-formal aspects of education [73–75], such as schooling, parental interactions, media, recreational activities, religious beliefs, and orientations, among others, thereby guiding behaviors and attitudes towards nature [3, 6, 7, 10, 13, 17, 30].

## Conclusions

Despite the predominance of wild vertebrates cited by the students of the present study, the frequent citations of domestic animals and invertebrates, and much more so among rural students, reflects, to a certain extent, conceptual misunderstanding about wild vertebrates. Furthermore, it is evidence of a limitation to the efficiency of the processes of formal education that address the theme. This finding may have repercussions both for future learning and for the development of attitudes towards wildlife and their conservation considering that in order to conserve one needs to know what passes for the basic conceptual notions.

Among the animals cited, the richest groups observed were mammals and reptiles, while the most diverse were mammals, birds, and invertebrates. The tendency for humans to identify mammals and birds has been observed by several studies, suggesting influences of phylogenetic proximity and/or utilitarian, esthetic, and behavior aspects, among others, convergent with the development of interest and affection for these animals, which we also consider for explaining, in part, the richness observed for reptiles.

The citation frequency for exotic animals, as well as that for other animals unlikely to be in the direct experience of students, is, in our opinion, an important aspect to consider. If knowledge of fauna external to the context of the life of students is not acquired concomitantly with knowledge of the local fauna and its ecological importance, there will not be a contribution to the expansion of knowledge in the local-global dimensions. Furthermore, and most importantly, their comparisons will contribute very little to the development of conservationist attitudes at the local level.

The tendency to recognize exotic animals is in line with another important conclusion of our study; “media” was indicated by the students as the main source of knowledge about animals, thus legitimizing the importance of contemporary media in knowledge about nature. In the current context, the possibilities of media interaction enhancing the exploration of the most diverse environments optimize individual knowledge of exotic animals. Furthermore, the prevalence of decontextualized formal educational processes in approaches to animal studies is seen to be incompatible with the prioritization of the immediate environment, which includes its fauna and its conservation.

Among the socioeconomic variables with potential influence on the understanding of “wild vertebrates,” the negative correlations between age and citations of domestic animals and invertebrates have led us to conclude that these were a consequence of diverse sociocultural influences, such as schooling, throughout individual development. It was also documented that the male gender recognizes greater animal diversity than the female gender, a tendency that has also been documented by several other studies. The data did not reveal influences by income on the citation of “wild vertebrates” by the students, since the correlation between these factors was weak. Likewise, religious orientation did not influence the citation frequency of wild vertebrates by the students. Finally, the positive correlation between curricular development and citation of wild vertebrates, as well as negative correlations between curricular development and citations of domestic animals and invertebrates, allows us to conclude that this is a cumulative consequence of the approach of the biological curriculum.

As far as the origin of knowledge about animals is concerned, the analyses revealed that “media,” “daily experiences,” “tradition,” and “formal education” stood out, respectively, in terms of citation frequency. The fact that the latter does not overlap, in terms of citation frequency in relation to the other categories, reflects, in our view, the impact and/or repercussion that schooling has on the lives of people in the contemporary context, marked by other possibilities of access to information, among which the media is perhaps the most representative, as has been observed elsewhere and in our study. Allied to this, it is necessary to consider that the prevalent curriculum practiced, which is based on rationalistic-academic “reproductivism,” is disconnected from reality in a double sense—it does not dialog with the biological content of the context of life, as expressed in “daily experiences” and “tradition,” and does not adequately appropriate the didactic efficiency of contemporary technological tools (e.g., media), it is a teaching-learning process.

However, in view of the multiplicity of information and knowledge accessible in contemporary times, we are bound by the idea that, although distinct, in practical life the categories of origin of knowledge about fauna defined in our study are not exclusive; that is, they interpolate and complement each other in reading, interpretation, and expression about life in all its dimensions.

## Footnotes

1 As a notion of diversity we take the measure of variability among individuals in a given context (e.g., large vertebrate groups addressed in this study—fish, amphibians, reptiles, birds, and mammals), considering the relative frequency of each type of animal cited. The more equal the frequency of citations of each individual type in the group, the greater the diversity; we consider the notion of “local diversity” to mean the number of species in a small area of homogeneous habitat [76].
